# Hybrid Whole-Genome Sequencing for Genetic Stability Assessment of Infectious Laryngotracheitis Virus Vaccine Strains

**DOI:** 10.3390/vaccines14030245

**Published:** 2026-03-07

**Authors:** Hee-young Jeong, Jessica Hicks, Su-min Go, Jin-ju Nah, Il Jang

**Affiliations:** 1 Veterinary Drugs and Biologics Division, Animal and Plant Quarantine Agency (APQA), Gimcheon-si 39660, Gyengsangbuk-do, Republic of Korea; 2 National Veterinary Services Laboratories (NVSL), USDA, 1920 Dayton Avenue, Ames, IA 50010, USA

**Keywords:** infectious laryngotracheitis virus (ILTV), whole-genome sequencing (WGS), hybrid genome assembly, vaccine strains, genome stability, core genome analysis, phylogenetic analysis

## Abstract

Background: Genetic stability of live-attenuated infectious laryngotracheitis virus (ILTV) vaccines is essential for consistent efficacy and safety; however, marker-based assessments targeting partial genes are often insufficient given the virus’s large, structurally complex genome. The ILTV genome contains long internal inverted repeats (IRs) that can give rise to genomic isomers, complicating short-read assembly and accurate resolution of genome structure. Methods: To overcome these limitations, we used a hybrid whole-genome sequencing (WGS) strategy, combining Oxford Nanopore Technologies (ONT) long reads to improve assembly contiguity with Illumina short reads for high-accuracy polishing at the single-nucleotide level. Using this approach, we generated complete de novo genome assemblies for the commercial Serva and Salsbury #146 vaccine strains. Results: The assemblies showed high sequence concordance with targeted regions validated by Sanger sequencing. Whole-genome analysis further enabled detection and independent validation of a structural inversion in the unique short (US) region of the Salsbury strain, consistent with herpesvirus genome isomerization. To enable phylogenetic inference despite structural variability, we performed a pangenome-based analysis to define a conserved core-genome dataset that robustly resolved vaccine-associated lineages, separating Serva- and Salsbury-derived strains. Conclusions: Collectively, these findings show that a hybrid WGS workflow can generate high-confidence genome assemblies for the specific commercial ILTV vaccine vials analyzed and can support QC-relevant detection of major structural variations. Because this study is cross-sectional (two strains; single lot/vial per strain), it cannot distinguish potential biological lot-to-lot variation from methodological differences, and a comprehensive genetic stability evaluation will require applying this workflow across defined passage levels and/or multiple production lots.

## 1. Introduction

Infectious laryngotracheitis (ILT) is a highly contagious respiratory disease of chickens caused by *Gallid alphaherpesvirus 1* (infectious laryngotracheitis virus; ILTV) and poses a major threat to the global poultry industry [1]. The disease is characterized by high morbidity and, in severe outbreaks, mortality that can reach 50% [2]. Rapid transmission and clinical similarity to other respiratory infections complicate early diagnosis and outbreak control [3], making ILT a persistent challenge to poultry health management worldwide [4].

Vaccination remains the primary tool for controlling ILT. Two major types of live-attenuated vaccines are used, namely chick-embryo-origin (CEO) and tissue-culture-origin (TCO) vaccines, which differ in their production history, administration route, and the immune response they elicit [5]. Despite their widespread use, vaccine performance can be compromised by genetic changes that accumulate during serial passage and/or following field circulation [6]. Therefore, a deeper understanding of the genomic features and genetic stability of ILTV strains is needed to ensure sustained vaccine effectiveness.

In Korea, the seed-lot system and the concept of genetic stability are applied to ensure vaccine quality and safety. Genetic stability requires that the genome sequence of a vaccine strain remain consistent across passages, and regulatory evaluation typically compares the master seed with the highest permitted passage level [7]. In this context, we selected two commercially available live-attenuated ILTV vaccines distributed in Korea and produced by worldwide manufacturers: Salsbury #146 (Zoetis) and Serva (MSD Animal Health). According to the manufacturers’ technical documentation, these vaccines were approved for use in South Korea in 1993 and 1998, respectively.

For many avian viruses, such assessments rely on sequencing a limited number of antigenicity-related marker genes—for example, the S1 gene of infectious bronchitis virus [8] or the F gene of Newcastle disease virus [9]. In such viruses, specific genotypic signatures associated with attenuation and/or phenotype can support targeted monitoring. Similarly, in the influenza virus, sets of attenuating mutations underlying live-attenuated vaccine backbones have been characterized, enabling stability assessments focused on defined loci and substitutions [10]. In contrast, ILTV possesses a relatively large double-stranded DNA genome of approximately 150 kb, and although multiple studies have explored candidate loci potentially associated with virulence attenuation (e.g., thymidine kinase [11], the major transcriptional regulator ICP4 [12], the non-essential gene UL0 [13], and several glycoproteins including gG, gJ, and gC) [14,15,16], these efforts have not yielded a universally accepted, standardized panel of key amino acid substitutions that consistently distinguishes vaccine strains from virulent field isolates across lineages and production histories. Given this multifactorial and context-dependent genetic basis, assessing ILTV vaccine stability using a single representative locus—or a small set of markers—remains inherently limited. Accordingly, whole-genome sequencing provides a more appropriate framework to capture genome-wide variation and to support high-resolution comparisons of vaccine seed lots across passages as part of genetic stability evaluation [17].

Beyond its multigenic determinants, the ILTV genome exhibits substantial structural complexity that limits what can be inferred from partial, locus-based sequencing. The genome is organized in a TRL–UL–IRL–IRS–US–TRS configuration, in which the unique short (US) region is flanked by inverted repeat regions (IRS and TRS) [18]. These repeats facilitate homologous recombination, which can invert the US region and generate multiple genomic isomers [6,19]. Because such rearrangements can occur independently of point mutations [20] and are not detectable from selected loci alone, partial sequencing may miss inversion events or region-specific variation, leading to incomplete or misleading conclusions about genetic stability. Together, ILTV’s multigenic basis and structural plasticity underscore the need for whole-genome sequencing to reliably evaluate vaccine strain stability [17,21]

Historically, ILTV whole-genome sequencing has relied on Sanger sequencing or short-read platforms such as Illumina [22]. However, Sanger sequencing is labor-intensive and time-consuming [23], and short reads alone often have difficulty resolving repeat-rich regions and reconstructing large, structurally complex herpesvirus genomes de novo [24]. Moreover, reference-based mapping can introduce reference bias and reduce sensitivity for detecting novel variants or structural changes [25,26]. Pangenome-based approaches provide a comprehensive representation of genomic content across strains [27,28] and can support more robust assessments of genetic diversity and stability [29], which is particularly important for structurally variable genomes such as ILTV.

In this study, we developed a rapid and reliable whole-genome assembly strategy for ILTV using a hybrid workflow integrating Nanopore long reads with high-accuracy Illumina short reads. We also implemented a phylogenetic analysis framework to better characterize commercial vaccine strains in the presence of structural variability. Overall, our findings support accurate identification and genetic stability assessment of commercial ILTV vaccine strains used in Korea. Because we analyzed only two vaccine strains and a single commercial vial/lot per strain without serial passages or seed-to-maximal passage comparisons, this work represents a baseline characterization and methodological proof-of-concept rather than a comprehensive longitudinal or regulatory stability evaluation.

## 2. Materials and Methods

### 2.1. Virus Strains and Viral DNA Extraction

This study used two commercially available live-attenuated ILTV vaccine strains from different manufacturers in Korea: Serva and Salsbury #146. Vaccine vials were stored at 4 °C until use.

Each vaccine sample was diluted to a final volume of 1 mL with distilled water prior to DNA extraction. Viral DNA was extracted using the TANBead Nucleic Acid Extraction Kit (TANBead, Taoyuan, Taiwan) on a Maelstrom 4810 automated extractor (TANBead, Taiwan). A modified protocol was applied using 200 µL of the diluted sample with 20 µL of proteinase K (instead of the recommended 300 µL sample with 10 µL proteinase K), and DNA was eluted in a final volume of 50 µL. DNA concentration was measured using a Qubit 4 Fluorometer with the Qubit™ 1X dsDNA HS Assay Kit (Thermo Fisher Scientific, Waltham, MA, USA).

### 2.2. Sequencing

Sequencing libraries were prepared according to platform-specific protocols. Library quality and fragment size distributions were assessed using a TapeStation 1000 (Agilent Technologies, Santa Clara, CA, USA) prior to sequencing.

For Illumina sequencing, libraries were prepared using the NEXTFLEX^®^ Rapid XP DNA-Seq Kit v2 (PerkinElmer, Waltham, MA, USA) on a Zephyr G3 NGS Workstation (Revity, Waltham, MA, USA) with 100–500 ng input DNA and five PCR cycles. Libraries were sequenced by a commercial provider on a NovaSeq X Plus platform (Illumina, San Diego, CA, USA) to generate 2 × 150 bp paired-end reads.

For Oxford Nanopore Technologies (ONT) sequencing, libraries were prepared using the Native Barcoding Kit 24 V14 (ONT, Oxford, UK) with 500 ng DNA per sample, following the manufacturer’s instructions. Barcoded libraries were pooled and sequenced in-house on a MinION device (ONT, UK) using FLO-MIN114 (R10.4.1) flow cells.

### 2.3. Genome Assembly

Illumina raw reads were quality-checked using FastQC v0.11.9 [30]. Adapter sequences and low-quality bases were removed using Trimmomatic v0.39 [31], and trimmed reads were re-evaluated with FastQC. Host-derived reads were removed using Kraken2 v2.1.5 [32] with a host genome database, retaining viral reads for downstream analyses.

Oxford Nanopore Technologies (ONT) raw signals were base-called using Dorado v5.0.0 with the dna_r10.4.1_e8.2_400bps_sup model. ONT reads were taxonomically classified using Kraken2 v2.1.5 [32] with a custom database containing viral and host sequences, and reads assigned to viral taxa were extracted using extract_kraken_reads.py from KrakenTools [33]. The extracted viral reads were further filtered using Filtlong v0.2.1 [34], with a minimum read length of 5 kb to improve assembly quality. Read-length distribution metrics (median read length and read N50) for the Filtlong-filtered ONT reads were computed using NanoStat v1.6.0 [35]. Filtered ONT reads were assembled de novo using Canu v2.2 [36]. Resulting contigs were polished for three iterations using Pilon v1.24 [37] in combination with the vSNP_step1.py module from the vSNP pipeline [38], using host-filtered Illumina reads that were adapter- and quality-trimmed with Trimmomatic v0.39 [31]. Assembly quality and completeness were evaluated using QUAST v5.2.0 [39], with the ILTV reference genome (NC_075683.1) [40] used for comparative assessment. Sequencing coverage was estimated by mapping ONT reads to the polished assemblies using Minimap2 v2.30 [41], and read depth was calculated with SAMtools v1.19.2 [42].

To validate the final assemblies, each assembly was aligned against multiple complete ILTV reference genomes available in GenBank. The Serva assembly was compared with HQ630064.1 and KP677881.1 (laboratory-adapted Serva lineages), whereas the Salsbury #146 assembly was compared with KP677882.1 (Salsbury lineage reference). Regions showing discrepancies (e.g., small insertions, deletions, or inversions) were identified and subsequently confirmed by Sanger sequencing. PCR primers were designed to amplify approximately 250 bp flanking each target region on both sides, and bidirectional sequencing was performed to verify assembly accuracy, including junction-spanning PCR for the US-region inversion in Salsbury #146.

### 2.4. Pangenome Analysis

To assess gene content variation among ILTV strains, we performed a pangenome analysis using Panaroo v1.5.0. We used Prokka v1.14.6 to generate uniform GFF annotations across all ILTV genomes because Panaroo clustering is sensitive to inconsistent input annotations [43,44]. A core gene presence threshold of ≥95% (core + soft-core) was applied, and the resulting core genome alignment (MAFFT v7) [45] was used for downstream phylogenetic inference.

### 2.5. Phylogenetic Tree Inference and Visualization

Phylogenetic relationships were inferred from the CGA generated in Section 2.4. Maximum-likelihood phylogenetic analysis was performed using RAxML-NG v1.2.2 [46] under the GTR + GAMMA substitution model. Branch support was assessed using 300 bootstrap replicates. The final tree was visualized and annotated using iTOL v6 [47].

## 3. Results

### 3.1. Sequencing and Genome Assembly Statistics

In this study, two ILTV vaccine strains were sequenced using a hybrid strategy combining Illumina short reads and Oxford Nanopore Technologies (ONT) long reads. Illumina sequencing generated approximately 5 Gbp of short-read data per strain, whereas ONT sequencing produced 7.4 Gbp (Serva) and 12.0 Gbp (Salsbury #146) of long-read data.

The hybrid assembly leveraged long-read information to resolve repetitive and structurally complex regions, yielding a single-contig, complete genome assembly for each strain. Assembly statistics, including genome length, GC content, mean sequencing coverage, and ONT read-length metrics after Filtlong filtering (median read length and read N50), are summarized in Table 1. After three rounds of Pilon polishing, the base-level accuracy was improved. Compared with the corresponding reference genomes, the Serva assembly differed by 10 single-nucleotide polymorphisms (SNPs) and the Salsbury assembly by 2 SNPs, most of which were located in or adjacent to homopolymeric tracts.

The final genome assemblies have been deposited in GenBank under accession numbers PX499071 (Serva) and PX499072 (Salsbury #146). These assemblies provided the basis for subsequent analyses of genomic stability, structural rearrangements, and genetic diversity in ILTV.

### 3.2. Sanger Validation of Sequence Variations and Structural Rearrangements

Following hybrid de novo assembly, we examined the genomic structure and sequence features of the two ILTV vaccine strains (Serva and Salsbury #146). QUAST analysis indicated that the Serva assembly aligned across the full length of the Serva-lineage reference genome (HQ630064.1), yielding a genome fraction of 100.0%. In contrast, the Salsbury assembly showed a genome fraction of 91.6% when evaluated against HQ630064.1; this is consistent with lineage-specific differences and alignment ambiguity in repeat-associated regions, including the US-region inversion described below.

To verify sequence differences identified in the assemblies, we performed PCR amplification and Sanger sequencing of loci that differed from the corresponding reference genomes. For the Serva strain, all 43 validated loci matched the de novo contig (100% concordance; Supplementary Table S2). By comparison, concordance between the Sanger results and the reference sequences was lower (HQ630064.1: 46.2%; KP677881.1: 87.2%). Similarly, for Salsbury #146, the de novo contig showed 100% concordance with the Sanger results (Supplementary Table S3), exceeding the concordance observed between Sanger data and the KP677882.1 reference genome (85.7%).

For concordance assessment, positions showing degenerate Sanger patterns—most frequently within homopolymeric or low-complexity regions—were classified as low-confidence sites. These sites were considered concordant when the de novo assembly and the corresponding reference sequence exhibited the same indel pattern. Low-confidence sites and strain-specific validation summaries are provided in Supplementary Table S2 (Serva) and Supplementary Table S3 (Salsbury #146).

A summary of the accession numbers and concordance ratios across the assessed sequences is provided in Table 2.

**Figure 1:**
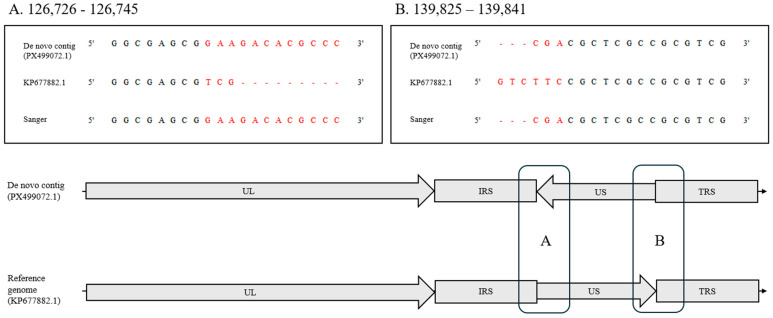
Structural inversion in the unique short (US) region of the ILTV Salsbury #146 strain. In panels (**A**,**B**), the sequences shown in red correspond to the US region. UL, unique long region; US, unique short region; IRS, internal repeat short region; TRS, terminal repeat short region. TRL and IRL are relatively short sequences and were omitted from the schematic representation for clarity.

In the Salsbury #146 strain, we identified a structural inversion within the US region flanked by the IRS and TRS repeats. The KP677882.1 reference genome represents one orientation, whereas the de novo assembly (PX499072) showed the opposite orientation. Sanger sequencing across both inversion junctions (positions 126,734 and 139,827 in PX499072) matched the de novo assembly sequence and differed from the reference orientation (Figure 1). These results validate the presence of a US-region inversion and support the structural accuracy of the hybrid assembly.

Collectively, these results support the reliability of the hybrid assembly and Sanger-validation workflow for resolving both nucleotide-level variation and structural rearrangements in complex herpesvirus genomes.

The inversion occurs within the US region flanked by the internal repeat short (IRS) and terminal repeat short (TRS) regions. UL, unique long; US, unique short. All coordinates are reported relative to the de novo contig PX499072. The inverted segment spans 126,734–139,827 in PX499072 and 126,736–139,829 in the reference genome KP677882.1.

### 3.3. Pangenome Analysis

To characterize variation in gene content and identify conserved genes among ILTV strains, we performed a pangenome analysis using Panaroo. The dataset included the two vaccine genomes assembled in this study together with all publicly available complete ILTV genomes, resulting in a pangenome of 153 genes across all strains.

Genes were classified by prevalence as follows: core genes (present in 99–100% of strains; *n* = 93), soft-core genes (present in 95–<99% of strains; *n* = 10), shell genes (present in 15–<95% of strains; *n* = 10), and cloud genes (present in <15% of strains; *n* = 40).

For downstream phylogenetic inference, the core genome alignment (CGA) was constructed from genes present in ≥95% of strains (core + soft-core; *n* = 103). We compared trees inferred using genes present in ≥99% of strains (*n* = 93) versus ≥95% of strains (n = 103; core + soft-core) and selected the ≥95% set because it yielded a topology more consistent with the strain information in Table S1. Focusing on this conserved genomic backbone provided a robust basis for resolving phylogenetic relationships and supporting genetic stability assessment of ILTV vaccine strains.

### 3.4. Core Genome-Based Phylogenetic Analysis

**Figure 2:**
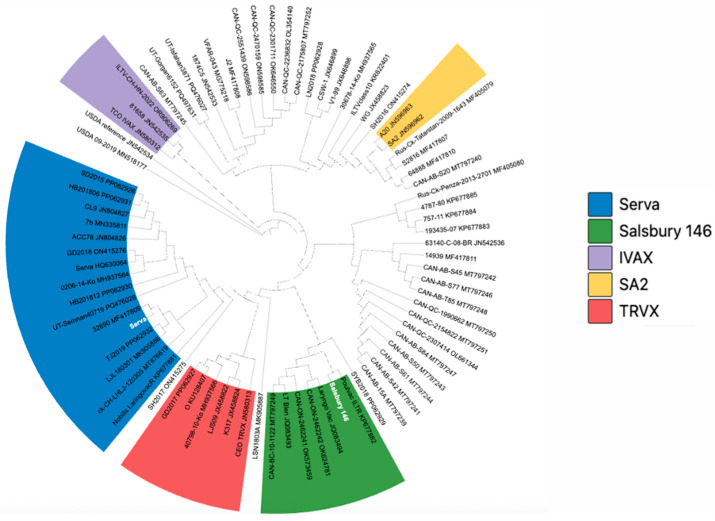
Core genome-based phylogeny of infectious laryngotracheitis virus (ILTV) strains. The core genome dataset was generated using Panaroo with a core gene presence threshold of ≥95% across strains. Maximum-likelihood phylogenetic inference was performed using RAxML-NG under the GTR+GAMMA substitution model, and the tree was visualized in iTOL. All major lineage-defining nodes have bootstrap support >90% (based on 300 replicates). GenBank reference genomes included in the dataset are highlighted in white. For clarity, the tree is displayed without branch-length scaling. Colored clades indicate major vaccine-associated lineages: Serva-related (blue), Salsbury #146-related (green), IVAX-related (purple), SA2-related (yellow), and TRVX-related (red).

Core genome-based phylogenetic analysis enabled clear clustering of ILTV strains and robust inference of relationships among major vaccine-derived lineages (Figure 2). Metadata for all GenBank genomes included in this analysis are provided in Supplementary Table S1.

Maximum-likelihood analysis separated the strains into five major groups that were consistent with their reported vaccine origins. Serva-related strains, including the de novo Serva assembly (PX499071), formed a well-supported Serva clade. This clade also included several field isolates (e.g., SD2015 and ACC78) that have been described as recombinant viruses but show shared ancestry with the Serva-derived vaccine lineage [6,48].

The Salsbury #146 assembly (PX499072) clustered within the Salsbury group together with other Salsbury-derived commercial strains. The LT-Blen strain, historically associated with the Hudson lineage [49], was positioned within this broader Salsbury-related cluster, and strain JQ083494 appeared closely related, sharing a recent common ancestor. Other vaccine-associated lineages (TRVX-, SA2-, and IVAX-related strains) were resolved as distinct, non-overlapping groups. Within the IVAX-related group, strain 81658 clustered closely with ILTV-CH-HN-2022, indicating close genetic relatedness.

The consistent placement of the Serva and Salsbury assemblies within their respective vaccine lineages supports distinct phylogenetic origins for the two strains. Importantly, the core genome approach likely reduces topological artifacts arising from structural variation in the US region, providing a reliable framework for genomic classification and surveillance of vaccine-derived ILTV lineages in the field.

## 4. Discussion

Vaccination strategies have substantially reduced the global burden of ILT since the introduction of live-attenuated ILTV vaccines. However, the emergence of more virulent strains associated with recombination between vaccine viruses [50] highlights the need for ongoing surveillance and robust genetic stability assessment. These concerns have increased interest in whole-genome sequencing (WGS) for ILTV. Historically, WGS of ILTV was performed largely using short-read platforms, reflecting earlier limitations in long-read sequencing technologies. This study is cross-sectional and provides a baseline genomic characterization of two commercial ILTV vaccine strains. Longitudinal genetic stability across serial passages and/or multiple production lots was not assessed here and should be addressed in future work. Because a standardized set of universally accepted attenuation markers has not been established for ILTV across vaccine lineages and field isolates, marker-only stability assessment remains limited, supporting the use of genome-wide approaches.

Reliance on short-read data alone poses challenges for de novo assembly of ILTV, particularly because repetitive, near-identical regions can lead to scaffolding failures and fragmented assemblies [51]. To address these limitations, previous studies often used PCR-based Sanger sequencing for gap filling and validation [52,53] or applied reference-guided mapping [17,54]. While Sanger-based finishing increases labor and cost, reference-guided approaches can introduce reference bias and reduce sensitivity for detecting novel variants or structural changes. Accordingly, we adopted a hybrid assembly strategy integrating ONT long reads with Illumina short reads. ONT reads improved assembly contiguity, whereas Illumina reads enabled base-level polishing to increase accuracy. Notably, the long internal repeat region of the ILTV genome spans ~13.8 kbp [17]; therefore, selecting long reads >5 kb was intended to improve structural resolution by increasing the likelihood of obtaining reads that bridge repeat-associated regions.

After Pilon polishing, the number of single-nucleotide differences between the initial ONT-derived contigs and the final polished assemblies was low (10 and 2 differences for the two strains, respectively). This suggests that ONT data alone may be adequate for some applications that are less sensitive to residual base-level errors (e.g., broad phylogenetic placement). Nevertheless, because downstream applications such as vaccine genetic stability assessment require maximal accuracy to detect subtle changes across passages, we retained the hybrid workflow to generate high-confidence reference assemblies suitable for longitudinal comparison. From a practical QC perspective, these results suggest diminishing returns from increasing ONT depth beyond the level required to obtain a complete long-read assembly when Illumina polishing is available. Thus, for routine QC aimed at generating a high-confidence consensus genome and detecting major structural variation, a pragmatic approach may be to target sufficient post-filtered ONT coverage for structural resolution (on the order of ~10× in our setting) while relying on Illumina short reads for cost-effective base-level accuracy.

To evaluate assembly accuracy, we compared our de novo genomes with previously published reference sequences for the same vaccine strains (Serva: HQ630064.1 [22], KP677881.1 [17], and Salsbury #146; KP677882.1 [17]). Sanger sequencing of loci that differed from the reference sequences matched the de novo assemblies at the validated sites, supporting the accuracy of the hybrid workflow. Differences from published references may reflect batch-to-batch (lot-specific) variation among commercial vaccines and/or technical differences among sequencing and assembly approaches; these possibilities cannot be unambiguously distinguished with the available data. Notably, mixed base calls were observed at specific loci in the Sanger traces. This observation was consistent with the Integrative Genomics Viewer (IGV) [55] inspection after mapping Illumina reads to the final assemblies . Similar mixed-base signals have been reported in prior analyses of ILTV vaccine and field strains [17,56], suggesting that this feature is not unique to the strains examined here. Although the biological basis remains to be determined, such signals may reflect within-lot heterogeneity, co-circulating subpopulations, or technical artifacts arising from amplification and sequencing. From a QC perspective, these mixed-base signals may indicate sub-consensus diversity within a vial/lot. Because the present workflow focuses on generating a high-confidence consensus sequence, systematic quantification of minor variants was not performed; if needed, targeted deep sequencing and/or variant-calling analyses could be incorporated in future QC.

For the Salsbury #146 strain, we observed an orientation difference in the US region relative to the published reference, consistent with isomerization of herpesvirus genomes and prior expectations for ILTV [22]. Even though US-region inversions can confound whole-genome alignments and bias phylogenetic inference, some studies have relied on whole-genome alignments despite this structural variability [22]. To mitigate this issue, we used a pangenome-based approach to derive a conserved core-genome dataset for phylogenetic reconstruction. Using this core-genome alignment, strains from the literature and GenBank were resolved into distinct clades consistent with their reported vaccine lineages, supporting the utility of a core-genome strategy for comparing ILTV genomes with complex structural variation. We did not quantify the relative abundance of US-orientation isomers in this study, which will require dedicated orientation-specific assays and will be addressed in future work.

Because the present study is cross-sectional (single commercial lot/vial per strain) and does not include serial passage series or multiple lots, it does not quantify genetic stability across passage levels or manufacturing lots and cannot distinguish potential biological lot-to-lot variation from methodological differences. Accordingly, future work should apply this workflow to additional ILTV vaccine lots and defined passage levels and should investigate the implications of mixed-base signals for vaccine production and quality control.

## 5. Conclusions

In summary, our hybrid assembly-based WGS workflow yields high-confidence genome assemblies for the specific commercial ILTV vaccine vials analyzed here, including genomes containing complex repeats, and enables the detection of major structural variations.

In addition, the core-genome phylogenetic framework provides an approach for lineage assignment that reduces sensitivity to US-region inversions, supporting comparative genomics and vaccine quality control applications. Accordingly, the validated WGS workflow and baseline assemblies reported here provide a useful framework for future comparative studies—including broader comparisons with field isolates and, where available, integration with phenotypic data—to better refine attenuation-associated genomic signatures.

## Figures and Tables

**Table 1:** Hybrid genome assembly metrics for ILTV vaccine strains.

Strain	Length (bp)	Genome Fraction (%) *	Mean Sequencing Depth (X) **		Post-Filtlong Filtering	
			Illumina	ONT ***	Median ONT Read Length (kb)	ONT Read N50 (kb)
Serva	153,350	100.0	348.8	13.0	6.6	10.2
Salsbury #146	153,644	91.6	183.2	39.4	7.2	8.6

* Genome fraction (%) was calculated as the proportion of bases aligned to the reference genome HQ630064.1. ** Mean sequencing depth (×) was calculated by mapping reads to the final polished assemblies; values are rounded to one decimal place. *** Oxford Nanopore Technologies (ONT).

**Table 2:** Concordance of de novo assemblies and reference genomes with Sanger sequencing at validated variant sites.

Strain	Serva			Salsbury #146	
Accession Number	PX499071.1 ^A^	HQ630064.1	KP677881.1	PX499072.1 ^A^	KP677882.1
Ratio ^B^ (no. concordant/no. assessed)	39/39 ^C^	18/39 ^C^	34/39 ^C^	7/7 ^D^	6/7 ^D^

^A^ GenBank acce ssion number for the de novo genome assemblies generated in this study. ^B^ Concordance is shown as the number of variant sites matching the Sanger result divided by the total number of assessed sites (excluding sites that could not be evaluated by Sanger sequencing). ^C^ For Serva, Sanger sequencing indicated 12 low-confidence (degenerate) sites, primarily in homopolymeric or low-complexity regions. These sites were considered concordant when the assembly/reference and the Sanger result supported the same indel pattern. ^D^ For Salsbury #146, Sanger sequencing indicated 6 low-confidence (degenerate) sites, evaluated using the same criteria as in (C).

## Data Availability

The whole-genome sequencing (WGS) data supporting the findings of this study are publicly available in the GenBank database under accession numbers PX499071 and PX499072.

## Supplementary Materials

The following supporting information can be downloaded at https://www.mdpi.com/article/10.3390/vaccines14030245/s1. Table S1: Detailed information on the ILTV genomes available in NCBI GenBank (accessed on 15 July 2025) used in the pangenome analysis. Table S2: Sanger validation of sequence discrepancies in the ILTV Serva strain. Table S3: Sanger validation of sequence discrepancies in the ILTV Salsbury #146 strain.

## Abbreviations

CEO	Chicken embryo origin
ILT	Infectious laryngotracheitis
ILTV	Infectious laryngotracheitis virus
IR	Internal inverted repeat
ONT	Oxford Nanopore Technologies
PCR	Polymerase chain reaction
SNP	Single-nucleotide polymorphism
TCO	Tissue culture origin
UL	Unique long
US	Unique short
WGS	Whole-genome sequencing
